# Manufacturing of Polymeric Substrates with Copper Nanofillers through Laser Stereolithography Technique

**DOI:** 10.3390/polym10121325

**Published:** 2018-11-29

**Authors:** Ely Dannier V-Niño, Andrés Díaz Lantada, Quentin Lonne, Hugo Armando Estupiñán Durán, Enrique Mejía-Ospino, Gustavo Ramírez-Caballero, José Luis Endrino

**Affiliations:** 1 Departamento de Ingeniería Mecánica, Universidad Politécnica de Madrid, 28006 Madrid, Spain; deydannv@gmail.com; 2 School of Aerospace, Transport and Manufacturing, Cranfield University, Bedfordshire MK43 0AL, UK; quentin.lonne@cranfield.ac.uk (Q.L.); j.l.endrino@cranfield.ac.uk (J.L.E.); 3 Departamento de Materiales y Minerales, Universidad Nacional de Colombia, 050034 Medellín, Colombia; haestupinand@unal.edu.co; 4 Escuela de Química e Ingeniería Química, Universidad Industrial de Santander, 680002 Bucaramanga, Colombia; emejia@uis.edu.co (E.M.-O.); gusramca@uis.edu.co (G.R.-C.); 5 Materials Science and Technology Research Group, Foundation of Researchers in Science and Technology of Materials, 680003 Bucaramanga, Colombia

**Keywords:** nanowires, mass-functionalization, additive manufacturing, laser stereolithography

## Abstract

This study presents the additive manufacture of objects using mass-functionalized photo-resins, which are additively photopolymerized using the laser stereolithography technique. The mass functionalization is based on the incorporation of copper nanowires used as fillers at different concentrations. Cylindrical and tensile test probes are designed and manufactured in a layer-by-layer approach using a low-cost laser stereolithography system working with a layer thickness of 100 µm. The morphological, mechanical, thermal and chemical results help to show the viability and potential that this combination of mass-functionalized resins and technological processes may have in the near future, once key challenges are solved. Finally, some potential applications are also discussed.

## 1. Introduction

In traditional manufacturing methods, typically based on subtractive approaches, the objects are manufactured by eliminating material from a preform with limited geometrical complexity, according to the established requirements, and usually generating a lot of waste. In additive manufacturing processes (commonly called 3D printing), the object is constructed in a more controlled form, using only the exact amount of material, which is deposited layer by layer, following a design obtained with a CAD program (computer-aided design) [1,2]. In a way, the straightforward solid free-form fabrication of complex objects and the sustainable production without much debris and in a wide set of materials are promoted. For these reasons, additive technologies are reinventing production processes and enabling the rapid production of innovative products and complex geometries, thanks to the layer-by-layer approach, with a degree of complexity that cannot be matched by more traditional methods and techniques, although the final performance of manufactured parts is not always as perfect as that obtained with traditional procedures [1,2,3,4,5]. Consequently, finding alternative methods that allow improving the mechanical, thermal and electrical properties of materials employed in additive manufacturing is a relevant field of study with industrial and scientific significance. Both surface and bulk functionalization of the basic materials may lead to final devices with enhanced properties and innovative functionalities. Laser stereolithography (SLA) [6,7,8] is an industrial gold standard, among additive manufacturing techniques, used for the manufacture of devices by photopolymerization, which allows for a great control of the dimensions and features of the substrate, when compared to other additive manufacturing techniques [5,9,10,11,12], although final parts are sometimes too brittle for demanding industrial applications. In this order of ideas, this work focuses on designing and manufacturing substrates with enhanced functionalities and performance, thanks to systematically modifying the base material (commercial photo-resin for laser stereolithography) using copper nanowires (Cu NWs) as additives for mass/bulk-functionalization. The methods and materials used in the design and manufacture of the selected substrates and probes are described. Besides this, the analysis and discussion of the preliminary testing and evaluation results, obtained from morphological, mechanical, thermal and chemical characterization, performed upon the manufactured test geometries and probes, provide useful information regarding the potential of the presented approach. Finally, with the described experiments, we seek to validate an approach based on mass-functionalization for solving current challenges in the performance of parts obtained by additive manufacturing methods, especially in regards to improving the properties of devices obtained by laser stereolithography.

## 2. Experimental Details

### 2.1. Materials and Methods

**Figure 1:**
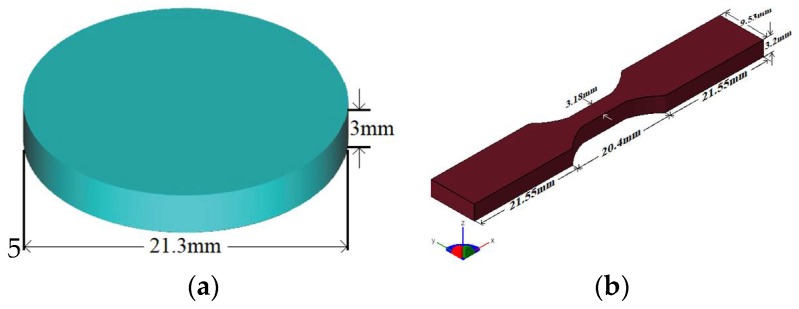
Testing substrates designed in FreeCAD. (**a**) Cylindrical geometry and (**b**) test probe for tensile testing (ASTM D638-14 type V).

The fabrication of the substrates for testing, including cylindrically shaped ones (21.3 mm in diameter and 3 mm thick) and tensile testing shaped ones (designed according to standard ASTM D638-14 type V [13]), starts with computer modeling using the FreeCAD v0.16 software. The CAD designs are presented in Figure 1. Subsequently, the CAD designs are exported to STL format (Standard Tessellation Language/STereoLithography), which is a sort of universal file format for additive manufacturing technologies and 3D printers.

**Figure 2:**
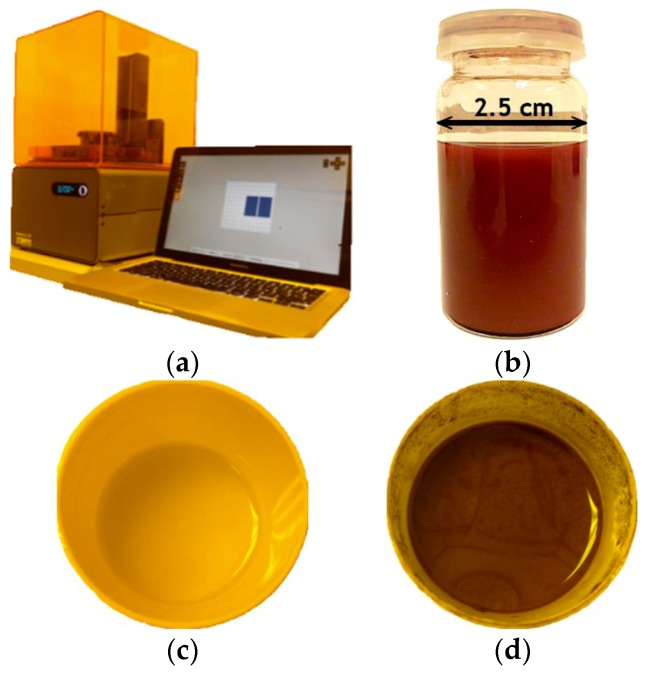
(**a**) Form 1+ printer. (**b**) Cu NWs in IPA. Resin types used in the substrates manufacturing. (**c**) Resin without Cu NWs and (**d**) Resin mixed with Cu NWs at 10.0% w/w.

The base material used for the manufacture of these devices is a commercial photoreactive resin commercialized under name “clear FLGPCL 02” (Form 1+, Formlabs) [14], which is a mixture of methacrylic acids esters (methacrylated oligomers and monomers) and photoinitiators, according to the manufacturer’s datasheet. The fabrication of the substrates with and without copper nanowires is carried out using the laser stereolithography technique, more specifically employing a Formlabs Form 1+ printer (see Figure 2a) [15], which is capable of reading the information of part geometries from the original CAD files exported to STL format. Laser stereolithography is a process working upon a liquid resin, whose polymerization is selectively activated by an ultraviolet laser. This laser gradually draws layers on the surface of the liquid resin, following a pattern defined in the STL file, after adequate slicing for layer-by-layer pattern and laser movement definition [5,12].

The copper nanowires (Cu NWs), employed for mass-functionalization, are provided by Surface Engineering & Precision Institute (Cranfield University, Bedford, UK) according to data reported in Table 1 and Figure 2b [16]. The mixture of “clear FLGPCL 02” resin with the Cu NWs stored in isopropyl alcohol (IPA) is performed in the MPC 004ST vacuum casting machine (SLM Solutions, Lübeck, Germany), with a 30° of inclination (Figure 2c,d). The features of the mixtures and the manufacturing parameters for 3D printing are reported in Table 1.

### 2.2. Characterization Techniques

**Figure 3:**
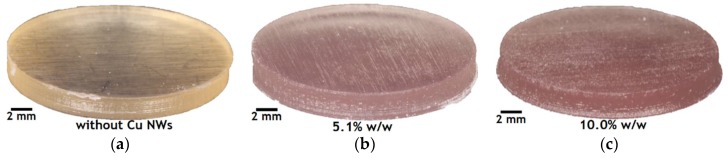
Manufactured circular substrates in the 3D Form 1+ printer. (**a**) without Cu NWs, (**b**) with Cu NWs at 5.1% w/w, and (**c**) 10.0% w/w.

**Figure 4:**
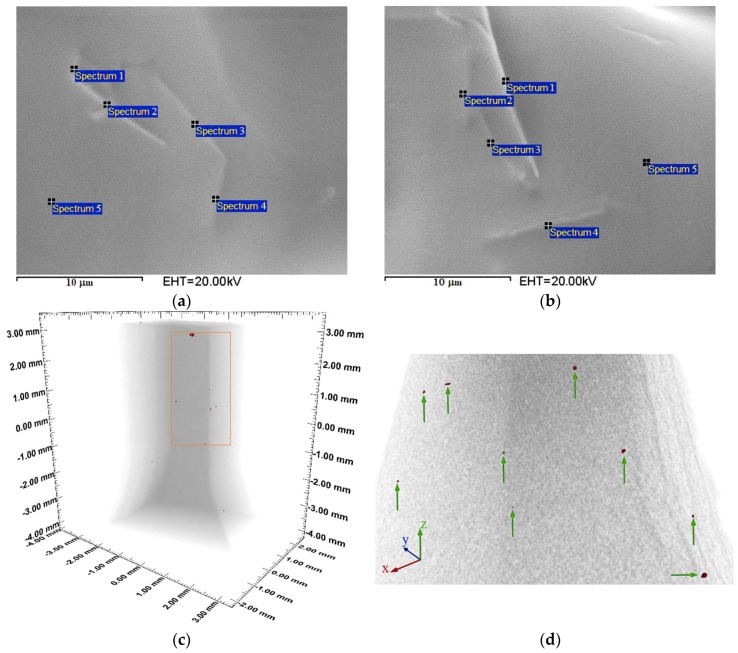
(**a**) Micrographs upon the built surface of a circular substrate with Cu NWs at 5.1% w/w. (**b**) Micrographs upon the built surface of a circular substrate with Cu NWs at 10.0% w/w. (**c**,**d**) 3D rendering of the substrate section that shows the Cu NWs distribution (rendered in red).

The surface morphology and presence of elements in the fabricated substrates with Cu NWs at 5.1% w/w and 10.0% w/w (Figure 3), are quantitatively and qualitatively analyzed by scanning electron microscopy (SEM) with a filament of tungsten (Figure 4a,b) and by energy dispersive spectroscopy (EDS) to 20 kV. Besides, with the purpose of identifying and understanding the Cu NWs distribution after manufacture, we perform a 3D rendering of the substrate with Cu NWs by using a computed tomography system XT H 160 CT SCAN, NIKON (Nikon, Tokyo, Japan). The beam energy used is 150 kV and 50 μA and the distance between the specimen and the detector is set to provide a degree of phase contrast to facilitate the visualization of nano-additives. The exposure time is set to 500 ms and 4 frames (each one of 540 projections) are collected by radiography. The voxel size is 10 μm, enough to image the notch region shown in Figure 4c,d.

**Figure 5:**
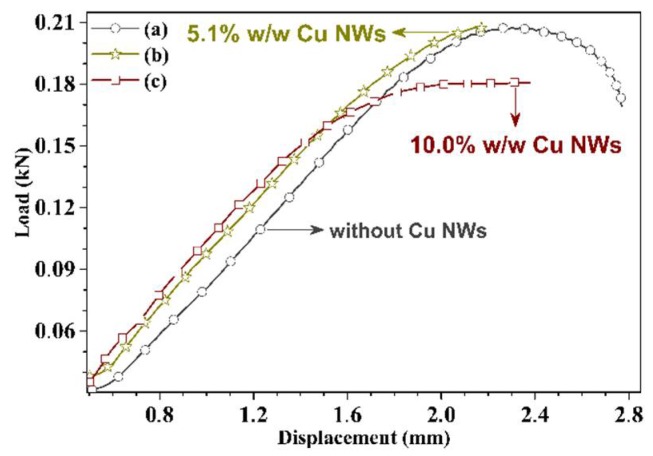
Representative load-displacement curves of specimens: (a) reference, resin without Cu NWs (◦), (b) with Cu NWs at 5.1% w/w (∗), and 10.0% w/w (□).

**Figure 6:**
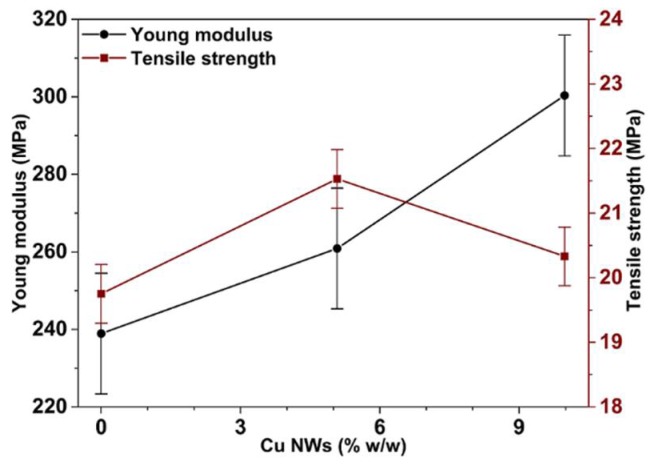
Effect of Cu NWs content on Young’s modulus (•) and tensile strength (■).

**Figure 7:**
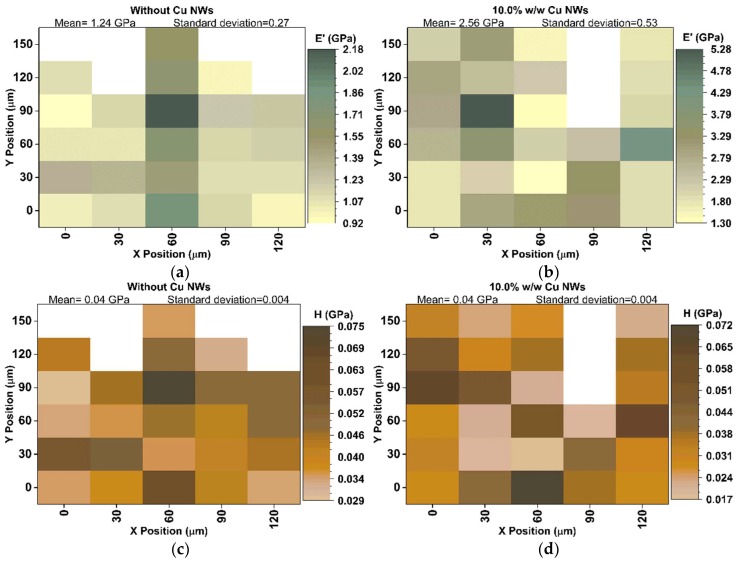
Contour maps of mechanical properties variation in the measurements of $E^{\prime }$ (**a**,**b**) and H (**c**,**d**), obtained on a surface area of 120 μm×150 μm in the substrates manufactured of resin without Cu NWs (**a**,**c**) and with Cu NWs at 10.0% w/w (**b**,**d**), respectively.

**Figure 8:**
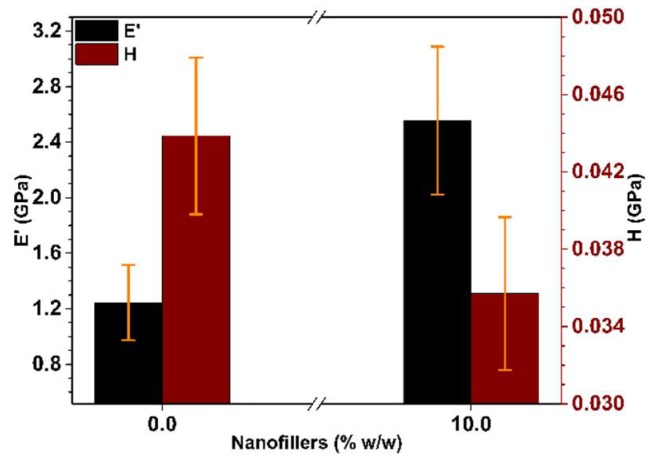
Effect of Cu NWs content on mechanical properties variation: $E^{\prime }$ storage modulus (Black) and H hardness (Red).

The mechanical properties are obtained through tensile tests and by instrumented nano-indentation. The tensile tests were carried out according to ASTM D638-14 standard [13], with type V specimens and a deformation speed of 1mm/min on an MTS model 835 system for testing universal. The Young’s moduli detected in the substrates are reported in Figure 5 and Figure 6. On the other hand, the reduced moduli ($E^{\prime }$) and hardness (H), are obtained of the complete cycles of loading and unloading in 30 indentations separated from each other 30 μm (Figure 7 and Figure 8). These tests are performed with a force of 10 mN and under 10 s creep, using the IBIS-Authority nano-indentation system of Fischer-Cripps Laboratory Pty Ltd. and with a Berkovich type indenter.

The behavior of dynamic mechanical analysis (DMA) of viscoelastic solids is carried out on a Q800 analyzer (TA Instruments Inc., New Castle, DE, USA). The experiment is conducted with the tension clamp, which clamps the sample at both ends, in the ramp/freq sweep test, to a frequency of 1 Hz, with a ramp rate of 5°C/min, in the range of temperatures from 30 to 130 °C and using force of 1 N. The instrument is completely calibrated in accordance with the procedures by TA Instruments.

**Figure 9:**
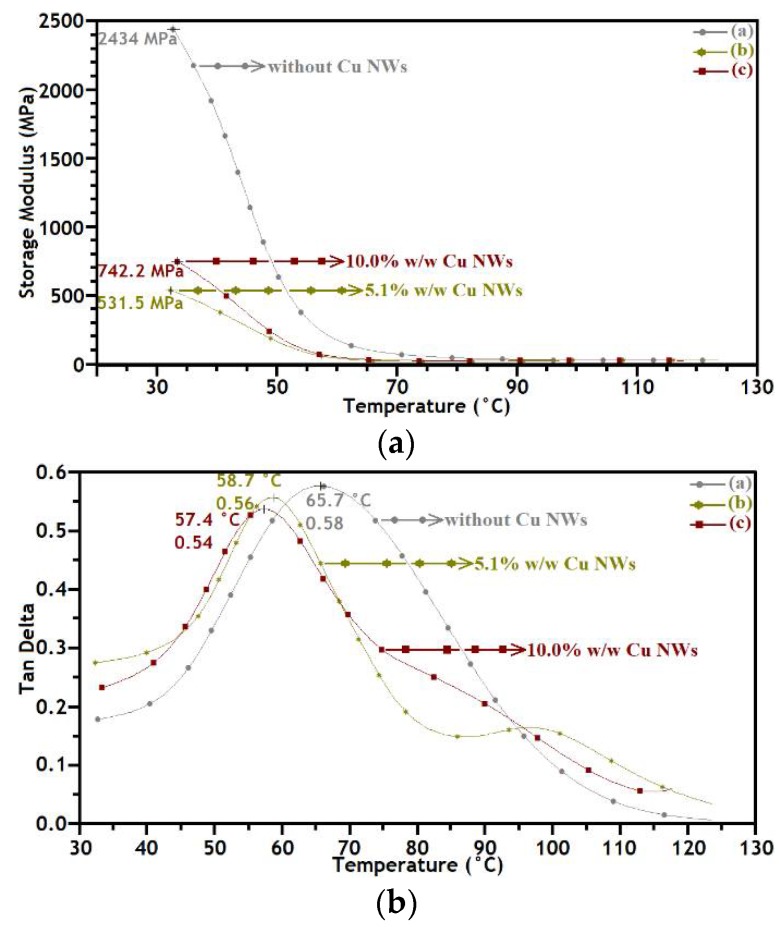
DMA curves of the substrates manufactured of “clear FLGPCL 02” resin without Cu NWs (◦), with Cu NWs at 5.1% w/w (∗), and 10.0% w/w (□). (**a**) Storage modulus ($E^{\prime }$) and (**b**) loss factor (tanδ) as a function of temperature.

In these tests: $E^{\prime }$ is the storage modulus, the elastic component that measures the energy stored during one oscillation cycle and that is related to the sample stiffness; $E^{\prime \prime }$ is the loss modulus, the viscous component the measures the mechanical energy dissipated through molecular motion in an oscillation cycle; and tanδ is the relationship between the elastic and inelastic component (phase lag referred to as loss tangent) that arises from any of the several molecular-level lossy processes such as entanglement, slip or friction between the monomers (Figure 9). In addition, tanδ reaches characteristic values of approximately of 1 for amorphous polymers in the transition zone, while it reaches values of around 0.1 for glassy and crystalline polymers [17].

**Figure 10:**
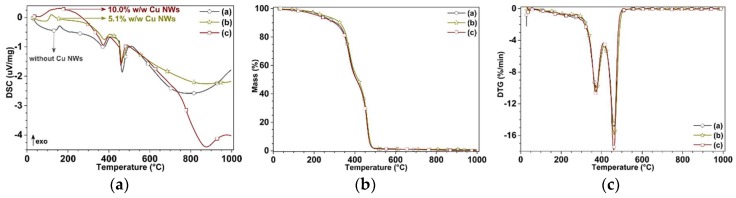
Thermal analysis of the substrates without Cu NWs (◦), with 5.1% w/w (∗), and with 10.0% w/w (□) Cu NWs. Representative curves (**a**) DSC, (**b**) TGA and (**c**) DTGA as a function of temperature.

The study of the thermal behavior of the substrates, manufactured with and without Cu NWs, is performed using differential scanning calorimetry (DSC) and thermogravimetric analysis (TGA) employing a STA 449 F5 Jupiter (NETZSCH, Selb, Germany), system operated under a nitrogen atmosphere for this purpose. Samples are analyzed between 32 and 100 °C with a heating rate of 10°C/min, to reveal their glass transition temperatures and structure-related information (see Figure 10).

**Figure 11:**
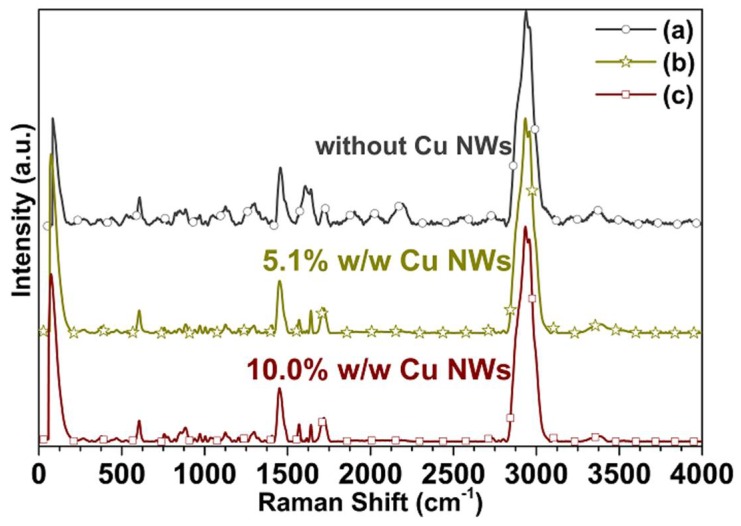
Raman spectra acquired on the substrates (a) without Cu NWs (◦), and with Cu NWs at (b) 5.1% w/w (∗), and (**c**) 10.0% w/w (□).

The characterization by Raman spectroscopy is performed, with the purpose to determine the vibrations and bands of present species on the surfaces of the substrates, by using the Horiba Scientific confocal spectrometer LabRam HR (HORIBA, Kyoto, Japan), which is equipped with a 532 nm laser and a 100X microscope objective. All Raman spectra are obtained with a 20 mW and a 15 mW laser power and a grating of 600g/mm (slit aperture) for 6 s and 8 s acquisition times (as shown in Figure 11). LabSpec 6 software Horiba Scientific (HORIBA, Kyoto, Japan), is used for Raman specters acquisition and analysis. Accordingly, each sample is scanned in the range of $25–4000{\;\mathrm{cm}}^{-1}$.

**Figure 12:**
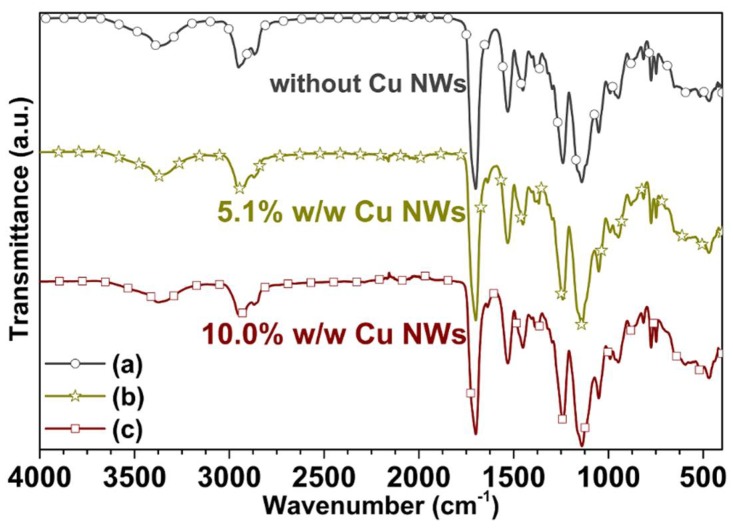
FTIR spectrums acquired on the substrates printed (a) without Cu NWs (◦), and with Cu NWs at (b) 5.1% w/w (∗), and (c) 10.0% w/w (□).

The Fourier-transform infrared (FTIR) spectra on substrates manufactured with and without copper nanowires is obtained by attenuated total reflection (ATR) technique in the Nicolet iS50 Spectrometer (Thermo Fisher Scientific, Waltham, MA, USA). The spectra of Figure 12 are analyzed in terms of transmittance in a wavenumber range from 400  to $4000{\;\mathrm{cm}}^{-1}$ using a resolution of $4{\;\mathrm{cm}}^{-1}$ and optical velocity of 0.1581cm/s. This analysis is used to determine the absorption bands of the organic functional groups on the surface of the substrates.

## 3. Results and Discussions

### 3.1. General Issues

Figure 3 shows the cylindrical substrates manufactured with and without Cu NWs at different concentrations (see Table 1), where the flat parallel surfaces (first and last manufactured layer) and their respective contours are well defined. However, it can be observed that the lateral surface of the substrates manufactured with Cu NWs, especially those of the one with a 10.0% w/w, present some irregularities or deformations, possibly because the laser with a 405 nm wavelength does not photopolymerize in perfect conditions, due to the presence of the additives. In principle, due to the high proportion of IPA that the Cu NWs solution contains, the curing time of each polymerized layer, whose superposition leads to the final object, should increase to compensate for the energy scattered due to the additives. Additionally, it is important to point out that the post-curing time, carried out at room temperature, should be longer when the IPA concentration increases, so as to compensate the lack of polymerization if the stereolithography machine’s working parameters cannot be modified, as is our case.

Table 2 reports the values of key measurements (length, mass and volume) of the cylindrical substrates, obtained from the CAD design and checked directly after manufacturing. The values show slight mismatches between the measurements obtained from the CAD file and from the actual prototypes (slightly higher). Furthermore, both the volumetric mismatch and mass decrease as the concentration of Cu NWs increases. It is likely that a significant part of the IPA contained in the resin evaporates during the 30-min mixing due to its high vapor pressure (43 mmHg at 20 °C). The rest may follow an esterification reaction with the methacrylic acids contained in the resin [18]. As the load of Cu NW solution increases in the resin, the samples prepared by SLA reach a lower final mass and volume. So, on the one hand, a higher content of Cu NW solution could lead to increased evaporation of IPA and to a higher decrease in mass (1.1% at 5.1% w/w and 4.6% at 10.0% w/w). On the other hand, a higher content of Cu NW solution would leave more residual IPA in the resin, leading to a higher rate of esterification, and hence to a more significant shrinkage of the polymer volume (3.7% at 5.1% w/w and 7.4% at 10.0% w/w) [19]. Finally, as the decrease in mass is more important than the decrease in volume, the density increases by 2.8% and 3.0%, when the load of Cu NW solution increases to 5.1% w/w and 10.0% w/w, respectively. This can be taken into account for design purposes if precise geometrical requirements upon final parts are needed.

The manufacturing time of a specimen, cylindrical or for tensile testing, with or without Cu NWs (see fabrication parameters in Table 1), is approximately 300 and 380 seconds respectively. Then, for the cylindrical substrate on average each layer, with a surface area of $3.5632{\;\mathrm{cm}}^{2}$, is manufactured in approximately 10 s. The cost of manufacturing a functional device with Cu NWs, depending on the percentage of Cu NWs added, increased approximately among a 25% and a 50%, when compared to the non-functional substrate. For instance, the cost of each specimen without Cu NWs is approximately 0.16 €, while that of those manufactured at 5.1% w/w and 10.0% w/w are 0.20 € and 0.22 € respectively. It is very important to keep in mind that this cost only considers the chemicals used for synthesis and washing of the NWs, and not the energy, the inert gas used and other complementary supplies for manufacturing. In Table 3 the approximate cost of materials used for each manufactured object or test probe is reported.

### 3.2. Morphology

The surface morphology and presence of elements in the fabricated substrates with Cu NWs at 5.1% w/w (Figure 3b and Figure 4a) and 10.0% w/w (Figure 3c and Figure 4b), are quantitatively and qualitatively analyzed by scanning electron microscopy (SEM) and energy dispersive spectroscopy (EDS). The micrographs in Figure 4a,b show well-dispersed Cu NWs in the photopolymerized resin. It is clear from these observations that the Cu NWs content is well below the percolation threshold. Consequently, the Cu NWs nanofillers are expected to influence the thermo-mechanical properties [20,21] of the polymer matrix, but not its electrical properties [22]. The spectra numbered with 1 to 4, in Figure 4a,b, are the regions where the Cu NWs were found. The spectra numbered with 5 are the regions without Cu NWs in both Figure 4a,b. Table 4 and Table 5 reports the identified elements, in atomic percent (At %), measured in the regions with and without Cu NWs. Additionally, according to the laser direction that polymerizes the resin during SLA printing, the surface roughness can vary significantly by adding the nano-additives and a homogenous filler distribution should be pursued to achieve an adequate dispersion for adequate manufacturability and functionalization (i.e., change of the mechanical and electromagnetic properties). In spite of possible improvements, the manufacturing of three-dimensional objects by laser stereolithography, using a Cu NWs mass-functionalized photopolymer, is demonstrated.

In Figure 4c,d, the X-ray computed tomography (CT) is used to capture the distribution and identification of the Cu NWs in the bulk of substrates manufactured in photoreactive resin through SLA technique by 3D printing. The brightness of X-ray CT images depends on the amount of X-ray penetration, which allows the identification of several Cu NWs randomly distributed into the bulk because, as the density of the specimens increases, the amount of X-ray penetration decreases, resulting in a brighter image [23,24,25]. In the substrate section analyzed, the bulk distribution of Cu nanowires is identified and corresponds approximately to a 0.003% of the $55.0{\;\mathrm{mm}}^{3}$ volume analyzed.

### 3.3. Mechanical and Thermal Performance

Representative load-displacement curves for the substrates manufactured using the photoreactive commercial resin, both with and without Cu NWs used for mass-functionalization, are provided in Figure 5. It can be observed that the curves without Cu NWs and with Cu NWs at 10.0% w/w have ductile behaviors, while the 5.1% w/w functionalization leads to a brittle behavior. Figure 5 and Figure 6 show that the tensile strength significantly increases in the specimen with Cu NWs at 5.1% w/w. Mechanical performance decreases in the substrate with Cu NWs at 10.0% w/w.

Tensile strength values are 21.5, 20.3 and 19.8 MPa for the specimen with Cu NWs contents of 5.1% w/w, 10.0% w/w and resin without nanofillers, respectively. For these samples, corresponding Young’s moduli are 260.9, 300.4 and 238.9 MPa as shown in Figure 6, respectively. The increase in mechanical performance is attributed to the successful load transfer from the matrix to the Cu NWs. The decrease in tensile strength above a Cu NWs content of 5.1% w/w may be related to the decrease in volume and mass when the content of Cu NWs increases, due to the IPA evaporation during cured and post-cured process. It could also be attributed to a decrease in the interfacial interactions between the polymeric matrix and fillers, due to solubility parameter that determined the substances affinity among the dissolvent and polymer, which do not differ in more than one or two units [26,27].

In addition, the presence of fillers may also affect the polymerization degree of the final device producing a plastification effect for the higher filler contents, which should always be taken into account in photopolymerization-based additive manufacturing techniques. Besides, during the strain of specimens, we determined that the molecular structure of the substrates without Cu NWs was the one capable of absorbing more energy, while the probe containing a Cu NWs at 5.1% w/w proved the worst in terms of energy absorbance. Furthermore, the specimens with Cu NWs at 5.1% w/w and 10.0% w/w showed more brittle fracture, when compared to the reference substrate.

Figure 7 shows the surface distribution of the values of storage or reduced modulus ($E^{\prime }$) and hardness (H), obtained by instrumented nano-indentation as explained in the materials and methods section. Figure 7 highlights that the variations in $E^{\prime }$ and H, when comparing the measurement performed on the substrates with Cu NWs and without Cu NWs, are not completely uniform. Figure 7 exhibits some areas with less and greater than the average deformation resistance values (see also Figure 8), in case of $E_{r}$ the minimal value acquired for Cu NWs at 10.0% w/w (Figure 7b) is major to the average value obtained in the pure resin (Figure 7a), while that the H values minimum, maximum and averages measured in the substrate surface with Cu NWs at 10.0% w/w are lower than obtained in the substrate without Cu NWs (Figure 8). In Figure 7, a strong correlation between $E^{\prime }$ and H is shown, where the areas probably reflect localized concentrations of Cu NWs than can be comparable in size to the hundreds of micrometer or nanometric-scales. Removal of such entangled agglomerates is a major focus of the many methods [28,29,30,31,32] used to disperse nanofillers in polymeric matrices. In general, when the agglomerates are only weakly infiltrated by the polymer, final properties of the composite are not as remarkable, as might be achieved by well-dispersed nano-additives bound to the matrix. This degradation of properties probably corresponds to the local “soft” spots, while the local “hard” spots probably reflect enhanced areas of Cu NWs concentration with adequate polymer infiltration (Figure 7). The advantage of such maps (Figure 7) is that local variations in properties are assessed directly and do not have to be inferred from measurements of entire composite components [31,32].

Figure 8 presents the average values of E’ and H for the pure resin and for the one loaded with the 10.0% w/w Cu NW solutions. The increase in storage modulus when loading the resin with Cu NWs can be once again attributed to the load transfer while the decrease in hardness could be due to the decrease of the density related to the evaporation of IPA during the UV curing of the resin.

Accordingly, if globally averaged properties are required for a polymeric or composite matrix to predict the overall response of a device, then the response of a number of indentations over a large enough area is required. Taking into account practical considerations, the indentation-based mapping of the mechanical properties of polymeric systems (soft materials) will always require indentation spacing greater than that employed for harder materials. Therefore, matching the indentation spacing and size to the length scale of the microstructure is important.

Figure 9 shows the change in $E^{\prime }$ and tanδ, for the substrates manufactured using the photo-resin without Cu NWs, with Cu NWs at 5.1% w/w, and 10.0% w/w, as a function of temperature. Figure 9a shows that $E^{\prime }$ of the substrate without Cu NWs has the highest value, 2434 MPa, and decreases with the addition of nanowires. The lowest value, 531.5 MPa, was obtained for the substrate with Cu NWs at 5.1% w/w. Accordingly, the factors such as the reaction degree and cross-linking density mainly influence the value of $E^{\prime }$. Besides this, the curves clearly highlight the glass transition region. Therefore, a distributed glass transition process in the material is confirmed, suggesting that the material behavior can be evaluated through rheological properties.

Figure 9b shows the change of tanδ of the substrates with Cu NWs with respect to those without Cu NWs. The peak value of tanδ is mainly influenced by the glass transition temperature ($T_{g}$). It can be appreciated that the increase of Cu nanowires, and the consequent lower cross-linking density, leads to higher mobility of polymer chains during glass transition and to a lower tanδ value. The sharper peak of tanδ indicates that a more regular structure is formed with the increasing content of Cu NWs. In these tests, the $T_{g}$ in the substrates without Cu NWs and with the loading of 5.1% w/w and 10.0% w/w reaches 65.7, 58.7, and 57.4 °C respectively.

The results of the DSC tests reported in Figure 10a, show for the first scan a broadly distributed second glass transition region and the presence of an exothermal signal. The distribution of the second glass transition region seems to cover regions: between 130–159 °C for the substrate without Cu NWs, between 90–119 °C for the substrate with 5.1% w/w Cu NWs and between 60−117 °C for the substrate with 10.0% w/w Cu NWs. Thus, the $T_{g}$ value slightly decreased as the Cu NWs loading in resin increased.

The thermal stability of the samples was investigated by TGA to ensure that the nano-additives are stable. Under pyrolytic conditions in the $N_{2}$ atmosphere, the degradation of the samples occurred with a sharp weight loss around 450 to 500 °C, accompanied with possible evolved organic fragments (e.g., methacrylic acid, ester, etc.) [33,34]. TGA results between 32 and 1000 °C are provided in Figure 10b. Slight mass loss from TGA curves, around 100 °C was attributed to the loss of absorbed IPA in resin, where most of the IPA to be evaporated during the UV post-curing, and moreover in general, between 100 and 200 °C, you lose the physisorbed water. Effects of Cu NWs on the thermal degradation behavior of the nanocomposites can be easily seen within the DTGA curves provided in Figure 10c. The thermal degradation temperatures ($T_{d}$), along with fusion temperature values ($T_{m}$) and glass transition temperature ($T_{g}$) of the substrates with and without nano-additives are tabulated and provided in Table 6. The fusion and degradation temperature are almost unchanged with the addition of the Cu NWs. One of the reasons for this behavior is the lack of chemical interaction between the resin and the Cu NWs. In fact, the degradation leads to a general breakage of C–C bonds, reducing the chemical cross-linking points and thus to an increase of the mobility of the polymeric chains which corresponds to a decrease of glass transition temperature. Accordingly, before the resin starts to degrade, surface interactions might get lost between the resin and the Cu NWs [34,35].

### 3.4. Spectroscopic Characterization

The Raman spectra obtained on the substrates manufactured in the “clear FLGPCL 02” resin without, with 5.1% w/w, and with 10.0% w/w Cu NWs are shown in Figure 11. The spectrum of Figure 11a reveals three Raman burly peaks at 1455 (strong), 2938 and $2956{\;\mathrm{cm}}^{-1}$ (very strong) that corresponded to the C−H bond vibrations, for the surface without Cu NWs. The peak (weak) at $607{\;\mathrm{cm}}^{-1}$ is attributed to the C−C bond vibration, and the peaks (medium) at 1606 and $1640{\;\mathrm{cm}}^{-1}$ are attributed to the C=C bond vibration. The band between 800 and $970{\;\mathrm{cm}}^{-1}$ correspond to the C−O−C bond vibrations, between 1300 and $1380{\;\mathrm{cm}}^{-1}$ to the $C-{\mathrm{CH}}_{3}$ bond vibrations, and between 1000 and $1150{\;\mathrm{cm}}^{-1}$ to the C−O−C bond asymmetric stretching vibrations. In $1404{\;\mathrm{cm}}^{-1}$ found the ${\mathrm{CH}}_{3}$ bond asymmetric stretching vibration and in $1723{\;\mathrm{cm}}^{-1}$ the C=O bond vibration. Additionally, the weak peaks at $2500-2850{\;\mathrm{cm}}^{-1}$, and $3140-4000{\;\mathrm{cm}}^{-1}$ are attributed to the $O-{\mathrm{CH}}_{3}$ and −H bonds vibrations respectively.

Raman spectroscopy reveals the surface interaction between polymeric organic materials and metallic Cu NWs. Consequently, the use of metal dopants such as the Cu NWs in the structure of organic materials allows, through surface-enhanced Raman scattering (SERS), may allow us to acquire the intrinsic vibrational fingerprint of photoreactive commercial resins more intense and defined bands, due to the extremely high sensitivity provided by plasmonic nanomaterials as such as the Cu, as is shown in Figure 11b,c [36,37,38]. In accordance with the above, this surface interaction can be responsible for the difference of $T_{g}$ before and after doping. Additionally, these studies allow us to identify differences in the degree of crystallinity due to the influence of the doping agents.

Figure 12 shows the FT-IR spectrum of the substrates manufactured using the photo-resin with and without nano-additives, where distinct absorption bands from 1150 to $1250{\;\mathrm{cm}}^{-1}$ appear, which can be attributed to the C−O−C stretching vibration. The two bands at 1386 and $748{\;\mathrm{cm}}^{-1}$ can be attributed to the α-methyl group vibrations. Moreover, the bands at 865, 1297, 1367 and $1405{\;\mathrm{cm}}^{-1}$ can be attributed to the C−C, $O-{\mathrm{CH}}_{2}$, C−H and C−OH stretching vibration, respectively. The band at $1700{\;\mathrm{cm}}^{-1}$ shows the presence of the ester group (C=O stretching vibration). The band at $1451{\;\mathrm{cm}}^{-1}$ can be attributed to the bending vibration of the C−H bonds of the $-{\mathrm{CH}}_{3}$ group. The two bands at 2952, 2932 and $2862{\;\mathrm{cm}}^{-1}$ can be assigned to the C−H bond stretching vibrations. Furthermore, there are two weak absorption bands at 3375 and $1637{\;\mathrm{cm}}^{-1}$, which can be attributed to the −OH group stretching and bending vibrations, respectively. Notwithstanding this, there are no appreciable differences between the FTIR spectra of the resin with and without Cu NWs, because the resin does not form any covalent bond with this type of nano-additives. Additionally, the Cu NWs do not add new functional groups that can be observed with infrared techniques (as appreciated from Figure 12b,c). Summarizing the above discussions, it can be concluded that the photoreactive polymer resin is macromolecular of type methacrylic acid (MAA) and methyl methacrylate (MMA) [35,39,40].

## 4. Conclusions

This study has focused on validating the manufacturability of three-dimensional objects using photopolymerizable resins mass-functionalized with copper nanowires by application of the layer-by-layer or additive manufacturing laser stereolithography technique. The manufacture of circular test geometries and tensile test probes made of Cu NWs within commercial photopolymer resin has been demonstrated. The effect of Cu NWs on the mechanical properties of the commercial photoreactive resin has also been characterized. Moreover, the mechanical properties of commercial resin were found to increase remarkably upon the addition of low proportions of Cu NWs. In our experiments, both the tensile strength and Young’s modulus values increased by around 10.0% with just a 5.1% w/w Cu NWs addition. As a result of this study, it is shown that Cu NWs nanocomposites have significant potential for different industrial applications, mainly for thermoset components with tuned mechanical properties. The mentioned potential may extend to other areas, which should be further analyzed, such as electrostatic packaging and MEMS, especially if future research leads to changes in electrical conductivity, together with the mechanical changes here described. Furthermore, the method described in this article is also suitable for decorative or functional applications, which may also benefit from possible improvements to the electrical properties, as just mentioned. Finally, we consider that this research work may be of interest for the 3D printing manufacturing community, especially for all those using the SLA technique, because it details a possible way of functionalizing polymeric resins with nano-additives and discusses the potentials and limitations of a wide set of characterization techniques for studying and evaluating the properties of photopolymerized composites.

## Figures and Tables

**Table 1:** Mass measurements of the Cu NWs and mixture and manufacturing features.

**Resin Type**	Clear FLGPCL 02			
**Type of additive**	Without Additive		Cu NWs	
**Mass of the nano-additive solution (g)**	N/A		6.0030	
**Nano-additive mass (g)**	N/A		9.8	
Additive percentage (% *w/w*) *	N/A		5.1	10.0
**Mixture time (min)**	N/A		30	30
**Substrate type**	Cylindrical	Tensile testing	Cylindrical	Tensile testing
**Quantity of printed substrates**	3	3	3	3
**Printing time (min)**	15	19	15	19
**Print resolution (µm)**	100	100	100	100
**Number of layers**	30	32	30	32

* Percentage weight per weight.

**Table 2:** Measurements of length, mass and volume of the cylindrical substrates.

Measurements from	Parameters	Substrate Type		
		Without Cu NWs	With Cu NW Sat 5.1 (% w/w)	With Cu NW Sat 10.0 (% w/w)
CAD design	Radius (cm)	1.065	1.065	1.065
	Thickness (cm)	0.300	0.300	0.300
	Volume ($cm^{3}$)	1.069	1.069	1.069
	Mass * (g)	1.176	1.176	1.176
Fabricated substrates	Radius (cm)	1.055	1.035	1.025
	Thickness (cm)	0.260	0.260	0.255
	Volume ($cm^{3}$)	0.909	0.875	0.842
	Mass (g)	1.017	1.006	0.970
	Density ($g/cm^{3}$)	1.119	1.150	1.152

* Values calculated with the density value of the resin as reported by manufacturer ($\rho =1.10\;g/{\mathrm{cm}}^{3}$).

**Table 3:** Cost of materials. * Considering just the chemicals used for synthesis and washing of NWs.

Materials	Cost
Liquid resin	0.13 (€/mL)
Cu NWs	0.45 (€/mg) *

**Table 4:** Detected elements in the surface with Cu NWs at 5.1% w/w.

Element	Spectrum 1	Spectrum 2	Spectrum 3	Spectrum 5
	At%	At%	At%	At%
C k	80.9	80.5	77.33	82.3
O k	17.7	18.4	21.8	17.7
Cu k	1.5	1.2	0.9	-

**Table 5:** Detected elements in the surface with Cu NWs at  10.0% w/w.

Element	Spectrum 1	Spectrum 3	Spectrum 4	Spectrum 5
	At%	At%	At%	At%
C k	82.5	76.9	81.3	81.7
O k	16.8	22.2	17.7	18.3
Cu k	0.7	0.9	1.0	-

**Table 6:** Values determined from DSC and TGA analysis.

Cu NWs Content	Transition and Degradation Temperatures (°C)		
	$T_{g}$	$T_{m}$	$T_{d}$
Without Cu NWs	159	374	465
5.1% w/w	119	378	462
10.0% w/w	117	372	458
